# Point prevalence and incidence of depressive and generalized anxiety symptoms among women attending antenatal clinics, a longitudinal study among adolescent mothers in Mwanza Tanzania

**DOI:** 10.1186/s13034-025-00983-5

**Published:** 2025-11-07

**Authors:** Matiko Mwita, Scott Patten, Deborah Dewey, Eveline T. Konje

**Affiliations:** 1 Psychiatry Department, Catholic University of Health and Allied Sciences (CUHAS), Mwanza, P. O. Box 1464, Tanzania; 2 Bugando Medical Centre (BMC), Psychiatry department, Mwanza, Tanzania; 3 Cuthbertson & Fisher Chair, Departments of Community Health Sciences and Psychiatry, University of Calgary, Calgary, Canada; 4 Departments of Pediatrics and Community Health Sciences, University of Calgary, Owerko Centre at the Alberta Children’s Hospital Research Institute, Hotchkiss Brain Institute, Calgary, Canada; 5 Department of Epidemiology and Biostatistics, Catholic University of Health and Allied Sciences (CUHAS), Mwanza, Tanzania

**Keywords:** Perinatal, Mental health, Depression, Anxiety, Adolescent pregnancy

## Abstract

**Background:**

Few studies have examined the point prevalence and incidence of perinatal mental disorders among the general population of pregnant women and no prior studies have investigated these in adolescent mothers. This study aimed to fill this gap by estimating the point prevalence and incidence of depressive and generalized anxiety symptoms, and their associated factors from pregnancy to 3 months post-delivery.

**Methods:**

A longitudinal study was conducted among 533 adolescent pregnant women in their second trimester. A convenience sampling strategy was used to recruit participants from preselected antenatal clinics in Mwanza, Tanzania from 5th August 2023 to 30th June 2024. We used the Edinburgh Postnatal Depression Scale (EPDS) to assess depressive symptoms and the Generalized Anxiety Disorder − 7 (GAD-7) scale to screen for generalized anxiety symptoms. The Screening was done in the second trimester of pregnancy (T1), the third trimester (T2), four weeks postpartum (T3) and three months post-delivery (T4).

**Results:**

The point prevalence of both depressive and generalized anxiety symptoms decreased from the second trimester of pregnancy (T1) to 3 months post-delivery (T4). The point prevalence of depressive symptoms fell from 20.64% (95% CI, 0.17–0.24) at T1 to 9.90% (95% CI, 0.07–0.12) at T4, while the point prevalence of generalized anxiety symptoms fell from 22.33% (95% CI, 0.19–0.26) at T1 to 10.48% (95% CI, 0.08–0.13) at T4. In contrast, the incidence of both depressive and anxiety symptoms increased from recruitment (T1) through to 3 months post-delivery. Specifically, the incidence of depressive symptoms rose from 9.00% (95% CI, 0.07–0.12) at T2 to 11.89% (95% CI, 0.07–0.12) at T4, while the incidence of generalized anxiety symptoms rose from 7.20% (95% CI, 0.05–0.10) at T2 to 10.81% (95% CI, 0.06–0.11) at T4. At all-time points, being classified as displaying depressive symptoms was highly associated with being classified as displaying symptoms of anxiety.

**Conclusions:**

There was an increase in incidence in depressive and anxiety symptoms in adolescent women from the second trimester of pregnancy to three months post-delivery, but a decrease in point prevalence. These findings support the importance of integrating mental health services into existing antenatal and postnatal care services for adolescent women.

## Background

Common maternal mental disorders such as depression and anxiety are the third leading causes of disease burden globally among women of reproductive age and are expected to rise to first place by 2030 [1, 2]. Globally, about 10% of pregnant women and 13% of women who have given birth experience a mental disorder [2]. Mental disorders are approximately three times more prevalent in low- and middle-income countries (LMICs) than in high-income countries (HICs) [3]. Perinatal depression and anxiety are major concerns as they pose health risks to both the mother and infant [4, 5] and these risks are increased among adolescent mothers [6].

Among the general population of perinatal women, some studies have found the risk of perinatal mental disorders to increase from the first to second trimester of pregnancy [7, 8] with a significant shift from subthreshold diagnoses during pregnancy to full diagnoses during the postpartum period [9]. Bennett et al. conducted a systematic review to determine the prevalence of depression during pregnancy and found it to increase from 7.4% during the first trimester to 12.8% during the second, and 12% during the third trimester [8]. Dad et al. in their systematic review on the epidemiology of antenatal depression in Africa found prevalence to be lower at first trimester (9.90%) and higher at second trimester (32.2%) [10]. In contrast, other studies have found the risk of perinatal mental disorders to decrease from pregnancy to postpartum. A study by Andersson et al. reported a prevalence of depression of 29.2% among women in Sweden during pregnancy, which decreased to 16.5% postpartum [9]. Among the general population of Mexican women, the incidence of depressive symptoms was reported to be 11.4% at six weeks and 9.0% at six months postdelivery [11].

Though some have found it difficult to differentiate women at high or low risk of specific perinatal depression trajectories [12], others have identified social and psychological risk factors as the most common predictors of a higher burden of depressive symptoms [13], with variations across different periods of onset of perinatal depression symptoms [14]. Adolescents may be more vulnerable to perinatal mental health problems than the general population of women and several social and psychological factors have been reported to be associated with this increased vulnerability. They include unplanned or unwanted pregnancies, pregnancies before marriage, poor partner relationships, and rejection by family. In addition, community stigma, uncertainty about the future, feelings of defeat and sadness related to the pregnancy, social isolation, and a lack of support have been associated with heightened risks of depression and anxiety during the perinatal period [15, 16].

The Mwanza region in Northwestern Tanzania is the second largest region in the country and Mwanza is one of the fastest growing city in Tanzania [17]. A previous study conducted in a tertiary hospital in Mwanza reported that the prevalence of postpartum depressive and anxiety symptoms were 25.39% and 37.31%, respectively among adult women [18]. Mwanza has a large population of adolescents and has one of the higher rates of adolescent pregnancies in the country at 27% [19]. Few services exist that support maternal mental health or the mental health of pregnant adolescents and at present, there is a global scarcity of research on the incidence of perinatal mental disorders, specifically depression and anxiety, among adolescent mothers. Understanding the point prevalence and incidence of perinatal mental disorders and their associated factors among adolescents is crucial for planning the delivery of mental health services to this population and developing appropriate interventions [20]. This study aims to address this knowledge gap by assessing the point prevalence and incidence of depressive and generalized anxiety symptoms, and their associated factors from pregnancy to 3 months post-delivery.

## Methods

### Study design and settings

We conducted a longitudinal study included adolescent pregnant women ≤ 19 years of age attending antenatal clinics (ANCs). This study was part of a prospective cohort study with an intervention component where adolescent women were recruited and followed from second trimester of pregnancy to three months post-delivery. Adolescent women were recruited during their second trimester of pregnancy, as this is the point in time when most women first come for antenatal care in Tanzania.

Three ANCs were purposively selected, two clinics from Nyamagana district (Makongoro and Butimba) and one from Ilemela district (Buzuruga). These ANCs were the largest in the city, government owned and operated, and provide both antenatal and postnatal care services including reproductive and child health (RCH). Each clinic saw approximately 500 perinatal women a month and about 30 were adolescent pregnant women [21]. There were no significant differences in the profiles of the participants from the three recruitment sites.

### Participants and recruitment methods

A convenience sampling strategy was used, whereby each consecutive adolescent pregnant woman who registered at the reception desk of the ANCs was approached. To ensure 80% power, a sample size requirement of 476 was estimated a priori using precision-based calculations considering a prevalence of 32.9% for depression [15] and an alpha value of 5%. To account for possible attrition, we planned to recruit at least 10% more, hence ending with a total of 533 participants. Participants were recruited and followed from 5th August 2023 to 30th June 2024. Eligible adolescent women who agreed to participate in this study signed a written consent form. For participants who were less than 18 years of age, we obtained their written assent, and they were given a consent document for their parents or legal guardians (e.g., husband) to sign prior to their participation in the study. Potential participants who were physically ill at the time of recruitment were not approached regarding participation in the study. The response rate was excellent with almost all participants approached providing their consent for participation in the study.

### Procedure

Adolescent pregnant women were invited by the trained research assistants to participate. Those who consented were interviewed and screened for depressive and/or anxiety symptoms at four timepoints: at recruitment in the second trimester of pregnancy (T1), in the third trimester (T2), four weeks postpartum (T3) and three months post-delivery (T4). All the interviews were conducted at the clinic and were done by the same interviewer to maintain consistency in questioning and reduce potential for bias. Any participant who screened positive for depressive and/or anxiety symptoms at T1, T2, or T3 was invited to participate in a non-pharmacological intervention involving Cognitive Behaviour Therapy (CBT). The intervention was offered from the second trimester of pregnancy to three months post-delivery. The intervention was delivered in group therapy sessions by trained nurse midwives monthly at antenatal or postnatal care visits.

### Instruments

Participants completed a sociodemographic questionnaire (i.e., age, education level, marital status, who they live with, employment status, if the pregnancy was planned or not, and if they encountered any form of partner violence), the Edinburg Postnatal Depression Scale (EPDS), and the Generalized Anxiety Disorder − 7 (GAD 7) scale. The EPDS, a 10-item scale, asks women to rate how they felt in the past 7 days. Each question has four possible responses that are scored from 0 to 3. The participant’s scores on the 10 items are summed and can range from 0 to 30. A score of eight and below is considered as depression unlikely, 9–11 depression possible, 12–13 high possibility of depression, 14 and higher probable depression. In this study, participants were classified as displaying symptoms of depression if they obtained a score of nine or above on EPDS [22]. While studies from high-income countries (HICs) indicate that an EPDS ≥ 9 is a reliable indicator for the development of perinatal depression [23], evidence from low- and middle-income countries (LMICs) suggests a threshold of ≥ 10 [24]. We adopted a cut-off of ≥ 9 to maximize case detection.

GAD-7 is a 7-scale item that asks how a woman has been bothered by specific anxiety symptoms over the past two weeks. Each question has four possible responses scored from 0 to 3. The scores are then summed and can range from 0 to 21. Scores are classified as no anxiety symptoms (a score of 0–4), mild anxiety (a score of 5–9), moderate anxiety (a score of 10–14), and severe anxiety (a score of 15–21). Participants were classified as displaying generalized anxiety symptoms if they scored five or above on GAD-7 [25]. Studies have demonstrated the scores of five and above on the GAD-7 are associated with an increased risk of developing generalized anxiety disorders among perinatal populations [26, 27].

Although the EPDS and the GAD-7 are self-reports, we read all the questions to participants. Both measures have good internal consistency, Cronbach’s α = 0.83 for EPDS [28] and Cronbach’s α = 0.82 for GAD-7 [29]. The EPDS and GAD-7 have been extensively used with pregnant and postnatal populations and employed in global population-based research [22, 30–32]. They have been utilized in various countries in Africa and translated into many languages including Swahili, the national language of Tanzania [29, 33, 34].

### Data management and analysis

Because there was minimal attrition, participants who were lost to follow up were not considered in the final analysis nor in the incidence calculations, as a result there was no missing data beyond attrition. Frequencies were calculated for categorical data and means and standard deviations for continuous variables. The point prevalences of depressive and generalized anxiety symptoms were defined and calculated as the percentages of adolescent women with depressive or generalized anxiety symptoms at each point of assessment (Times 1, 2, 3 and 4). Incidence referred to new cases of depressive or generalized anxiety symptoms occurring at Times 2, 3 and 4 among those without symptoms at the previous timepoint. Multivariable logistic regression was used to compare adolescent women with or without depressive and/or generalized anxiety symptoms on sociodemographic characteristics and the presence of the other condition (depressive or anxiety symptoms) at each time. Statistical analyses were conducted using STATA version 15 for Mac.

## Results

### Participants recruitment and follow ups

**Fig. 1:**
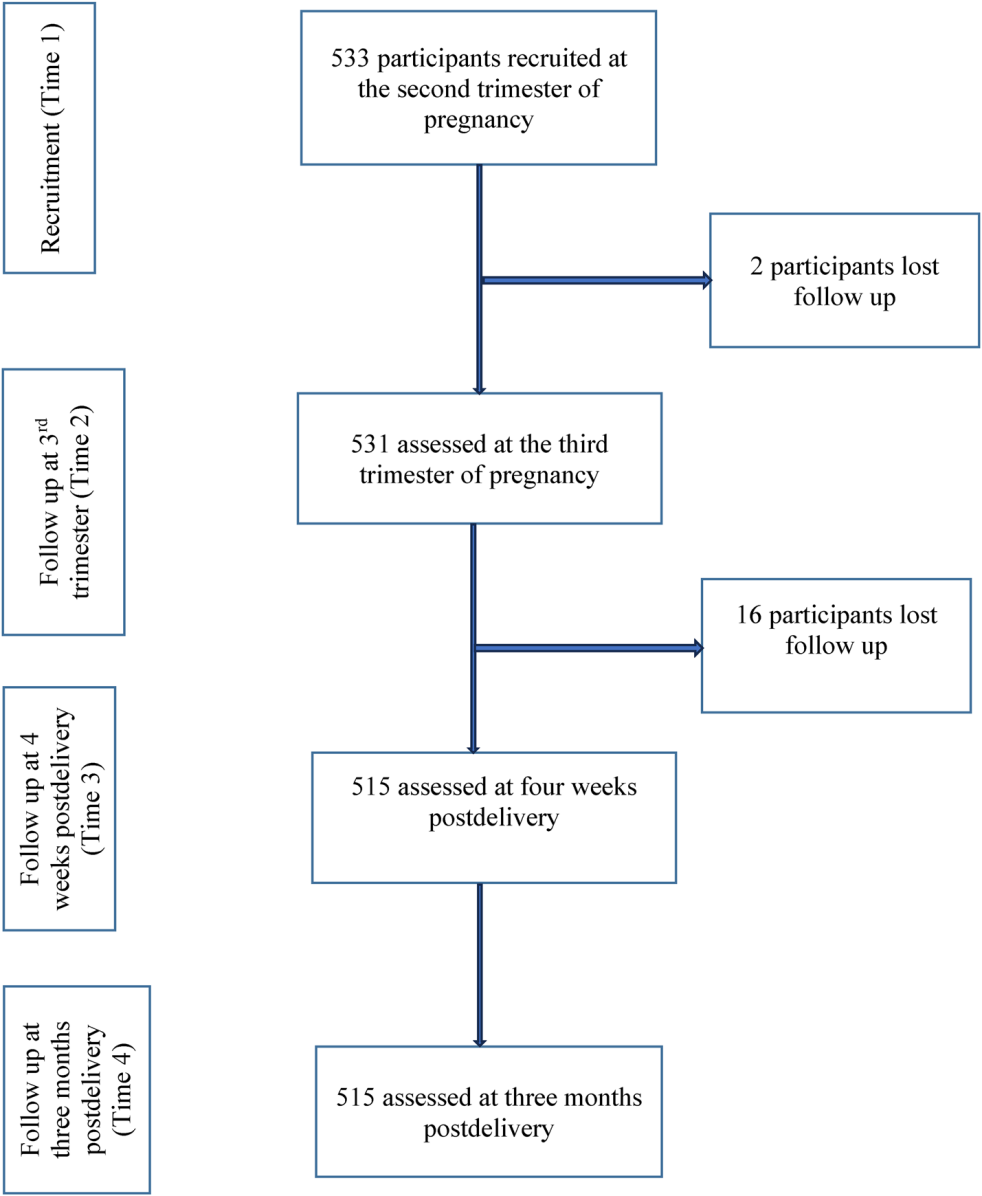
Flowchart of participants recruitment and follow ups

Overall, 533 participants were recruited and assessed at the second trimester of pregnancy (T1), of these 531 (99.6%) were assessed at T2, and 515 (96.6%) were assessed at T3 and T4. Eighteen participants (3.4%) were lost to follow up. Figure 1 elaborates the flowchart of participants recruitment and follow up.

### Sociodemographic and pregnancy related characteristics

At the time of recruitment, the mean age of the participants was 18.29 years (SD = 0.90; range 15–19 years); approximately half were 19 years of age, 52.72% (*n* = 281). The majority of participants were married and lived with their partners, 65.41% (*n* = 346). Most of the participants were prime-gravida, 88.56% (*n* = 472), and approximately half of the participants reported planning their pregnancy, 49.91% (*n* = 266). Table 1 summarizes the sociodemographic and obstetric characteristics of the study participants.

**Table 1:** Sociodemographic and pregnancy related characteristics of the study participants

Variable	Frequency (*n*)	Percentage (%)
**Age of pregnant adolescents (years)**		
< 18	91	17.07
18	161	30.21
19	281	52.72
**Marital status**		
Not married	183	34.33
Married	350	65.67
**Who do you live with?**		
With partner	353	66.23
With parents/caretakers	180	33.77
**Education level**		
Never went to school	31	5.86
Primary education	286	54.06
Secondary education	212	40.08
**Employment status**		
Never employed	444	83.30
Employed	89	16.70
**Pregnancy related factors**		
**Planned for the pregnancy**		
No	266	49.91
Yes	267	50.09
**Do you encounter any form of partner violence?**		
No	497	93.25
Yes	36	6.75

### Point prevalence and incidence of perinatal depressive and anxiety symptoms

The point prevalence of perinatal depressive symptoms (EPDS≥9) was 20.64% (95% CI, 0.17–0.24) in the second trimester of pregnancy (T1), 13.37% (95% CI, 0.11–0.16) in the third trimester of pregnancy (T2), 14.37% (95% CI, 0.11–0.17) at four weeks post-delivery (T3) and 9.90% (95% CI, 0.07–0.12) at three months post-delivery (T4). The incidence of perinatal depressive symptoms at T2 (*n* = 421) was 9.00% (95% CI, 0.07–0.12), at T3 (*n* = 367) it was 9.80% (95% CI, 0.06–0.12) and at T4 (*n* = 328) it was 11.89% (95% CI, 0.07–0.12) (Table 2). The observed incidence increased by 0.8% from T2 to T3 and by 2.09% from T3 to T4.

The point prevalence of generalized anxiety symptoms (GAD-7 ≥5) was 22.33% (95% CI, 0.19–0.26) in the second trimester of pregnancy (T1), 12.43% (95% CI, 0.11–0.15) in the third trimester of pregnancy (T2), 9.90% (95% CI, 0.07–0.12) at four weeks post-delivery (T3) and 10.48% (95% CI, 0.08–0.13) at three months post-delivery (T4). The incidence of generalized anxiety symptoms at T2 (*n* = 412) was 7.20% (95% CI, 0.05–0.10), at T3 (*n* = 366) it was 9.02% (95% CI, 0.06–0.11) and at T4 (*n* = 333) it was 10.81% (95% CI, 0.06–0.11) (Table 2). The observed incidence increased by 1.74% from T2 to T3 and by 1.79% from T3 to T4.

### Participation in a non-pharmacological intervention

A total of 373 participants who screened positive for depressive and/or anxiety symptoms at T1, T2, or T3 participated in a non-pharmacological intervention involving Cognitive Behaviour Therapy (CBT). The intervention was offered in group therapy sessions; total of 44 groups with eight to ten participants in each group were conducted. Six trained nurse midwives from the three ANCs delivered the interventions monthly at antenatal or postnatal care visits. Each group participated in at least five CBT sessions from recruitment to three months post-delivery.

**Table 2:** Point prevalence and incidence of depressive and anxiety symptoms

EPDS (≥9)	Time 1: 2nd trimester pregnancy *n* (%) 95%CI		Time 2: 3^rd^ trimester pregnancy *n* (%) 95%CI		Time 3: 4 weeks postdelivery *n* (%) 95%CI		Time 4: 3 months postdelivery *n* (%) 95%CI	
**Prevalence**	110 (20.64%)	0.17–0.24	71(13.37%)	0.11–0.16	74(14.37%)	0.11–0.17	51(9.90%)	0.07–0.12
**Incidence**	-	-	38/421(9.00%)	0.07–0.12	36/367(9.80%)	0.06–0.12	39/328(11.89%)	0.07–0.12
**GAD-7 (**≥5**)**								
**Prevalence**	119 (22.33%)	0.19–0.26	66(12.43%)	0.11–0.15	51(9.90%)	0.07–0.12	54(10.48%)	0.08–0.13
**Incidence**	-	-	30/412(7.28%)	0.05–0.10	33/366(9.02%)	0.06–0.11	36/333(10.81%)	0.06–0.11

### Sociodemographic differences among adolescent women with and without depressive symptoms

Associations between sociodemographic variables and depressive symptoms differed by time. In the second trimester of pregnancy (T1), older adolescents (OR = 0.4, 95% CI: 0.2,0.7, *p* = 0.001), those who were married (OR = 0.3, 95% CI: 0.2,0.5, *p* < 0.001), and those who planned for their pregnancy (OR = 0.2, 95% CI:0.1,0.3, *p* < 0.001) had significantly lower odds of depressive symptoms. Those living with parents/care takers (T1: OR = 3.8, 95% CI: 2.4,5.8, *p* < 0.001) had increased odds for depressive symptoms. Experiencing partner violence was associated with significantly increased odds for depressive symptoms in pregnancy and at two weeks post-delivery (T1: OR = 3.4 95 CI: 1.7,6.7, *p* = 0.001, T2: OR = 3.4, 95% CI: 1.6,7.5, *p* = 0.002, Time 3: OR = 2.5, 95% CI: 1.1,5.5, *p* = 0.026). At T4 (three months postpartum), those who were married showed significantly increased odds for depressive symptoms (OR = 7.2, 95% CI: 1.4,37.9, *p* = 0.020). Anxiety symptoms were highly associated with depressive symptoms at all time points (Time 1: OR = 35.8 95 CI: 20.5,62.7, *p* < 0.001, Time 2: OR = 12.3, 95% CI: 6.9,22.2, *p* < 0.001, Time 3: OR = 149.2, 95% CI: 54.9,404.9, *p* < 0.001, Time 4: OR = 101.2, 95% CI: 44.3,231.2, *p* < 0.001). Education level and employment status were not associated with depressive symptoms during pregnancy or at 3 months postpartum. Table 3 summarizes the association between depressive symptoms and sociodemographic factors during pregnancy and post-delivery.

**Table 3:** Associations between depressive symptoms and sociodemographic factors during pregnancy and post-delivery

Variables	Depressive symptoms Time 1: 2nd trimester Pregnancy		Multivariable Logistic Associations							
			Time 1: 2nd trimester Pregnancy		Time 2: 3rd trimester Pregnancy		Time 3: 4 weeks post delivery		Time 4: 3 months post delivery	
	No (*N* %)	Yes (*N* %)	OR (95% CI)	*P* value	OR (95% CI)	*P* value	OR (95% CI)	*P* value	OR (95% CI)	*P* value
19 years of age	236(83.99)	45(16.01)	0.4(0.2–0.7)	0.001	0.9(0.7–1.3)	0.923	1.1(0.7–1.9)	0.629	1.3(0.7–2.4)	0.368
Being Married	298(86.13)	48(13.87)	0.3(0.2–0.5)	< 0.001	0.7(0.2–2.9)	0.650	2.7(0.7–11.3)	0.169	7.2(1.4–37.9)	0.020
Living With parents/caretakers	120(64.17)	67(35.83)	3.8(2.4–5.8)	< 0.001	0.6(0.1–2.4)	0.464	2.5(0.6–10.1)	0.198	4.5(0.9–22.4)	0.066
≥ Secondary education	165(77.83)	47(22.17)	0.8(0.3–1.9)	0.652	0.9(0.5–1.5)	0.576	0.8(0.5–1.3)	0.365	0.9(0.5–1.7)	0.782
Employed	67(75.28)	22(24.72)	1.3(0.8–2.2)	0.343	1.0(0.4–2.2)	0.954	1.22(0.6–2.5)	0.588	1.2(0.5–2.8)	0.639
Planned pregnancy	240(90.91)	24(9.09)	0.2(0.1–0.3)	< 0.001	0.6(0.3–1.1)	0.079	0.6(0.4–1.2)	0.153	0.6(0.3–1.2)	0.141
Experiencing any form of partner violence	20(55.56)	16(44.44)	3.4(1.7–6.7)	0.001	3.4(1.6–7.5)	0.002	2.5(1.1–5.5)	0.026	1.5(0.5–4.3)	0.417
Generalized anxiety symptoms	35(29.41)	84(70.59)	35.8(20.5–62.7)	< 0.001	12.3(6.9–22.2)	< 0.001	149.2(54.9-404.9)	< 0.001	101.2(44.3-231.2)	< 0.001

### Sociodemographic differences among adolescent women with and without anxiety symptoms

Associations between sociodemographic variables and anxiety symptoms also differed by time (see Table 4). In the second trimester of pregnancy, (Time 1), those who were married (OR = 0.7, 95% CI: 0.1,0.6, *p* = 0.043), and those who planned for their pregnancies (OR = 0.4, 95% CI:0.3,0.8, *p* = 0.005) had significantly lower odds of anxiety symptoms. Those living with parents/care takers (OR = 10.3, 95% CI: 1.5,5.3, *p* = 0.016), had increased odds for anxiety symptoms. Experiencing partner violence was associated with significantly increased odds for anxiety symptoms during pregnancy (Time 1: OR = 4.6, 95% CI: 2.1,9.9, *p* < 0.001, Time 2: OR = 3.0, 95% CI: 1.3,6.8, *p* = 0.007). Those who planned for their pregnancies showed significantly decreased odds for anxiety symptoms post-delivery (Time 3: OR = 0.4, 95% CI: 0.2,0.8, *p* = 0.012, Time 4: OR = 0.5, 95% CI: 0.2,0.9, *p* = 0.022). At Time 4 (three months postpartum), those who were married (OR = 6.9, 95% CI: 1.4,3.5, *p* = 0.018) and those living with parents/care takers (OR = 4.9, 95% CI: 1.2,3.5, *p* = 0.047) showed significantly increased odds for anxiety symptoms. Age, education level and employment status were not associated with anxiety symptoms during pregnancy or post-delivery.

**Table 4:** Associations between generalized anxiety symptoms and sociodemographic factors during pregnancy and post-delivery

Variable	Generalized Anxiety symptoms Time 1: 2nd trimester Pregnancy		Multivariable Logistic Associations							
			Time 1: 2nd trimester Pregnancy		Time 2: 3rd trimester Pregnancy		Time 3: 4 weeks post delivery		Time 4: 3 months post delivery	
	No (*N* %)	Yes (*N* %)	OR (95% CI)	*P* value	OR (95% CI)	*P* value	OR (95% CI)	*P* value	OR (95% CI)	*P* value
19 years of age	229(82.37)	49(17.63)	0.7(0.4–1.2)	0.168	1.4(0.6–3.1)	0.456	1.3(0.7–2.3)	0.448	1.3(0.7–2.3)	0.424
Being Married	287(82.95)	59(17.05)	0.7(0.1–0.6)	0.043	4.2(0.9–18.7)	0.061	3.9(0.7–20.2)	0.097	6.9(1.4–3.5)	0.018
Living With parents/caretakers	123(65.78)	64(34.22)	10.3(1.5–5.3)	0.016	4.3(0.9–18.4)	0.052	2.7(0.6–13.6)	0.215	4.9(1.2–3.5)	0.047
≥ Secondary education	164(77.36)	48(22.64)	0.8(0.4-2.0)	0.696	0.7(0.4–1.2)	0.173	0.8(0.5–1.5)	0.473	0.9(0.5–1.8)	0.974
Employed	70(78.65)	19(21.35)	0.9(0.5–1.6)	0.776	0.9(0.4–1.9)	0.720	1.1(0.4–2.7)	0.842	1.4(0.6–3.2)	0.404
Planned pregnancy	232(87.88)	32(12.12)	0.4(0.3–0.8)	0.005	0.7(0.4–1.3)	0.242	0.4(0.2–0.8)	0.012	0.5(0.2–0.9)	0.022
Experiencing any form of partner violence	17(47.22)	19(52.78)	4.6(2.1–9.9)	< 0.001	3.0(1.3–6.8)	0.007	2.2(0.9–5.5)	0.086	2.3(0.9–5.8)	0.074

## Discussion

Our study found an increase in incidence of both depressive and anxiety symptoms in adolescent women from the second trimester of pregnancy to three months post-delivery, but a decrease in point prevalence. This study also revealed a strong association between depressive and anxiety symptoms in pregnant adolescent women. This was consistent with previous studies, that have reported high co-occurrence rates between these two disorders in the general population of adolescents [35] and in perinatal women [36, 37]. Overall, these findings provide new information on depression and anxiety symptoms among pregnant and postpartum adolescent women living in a low resource setting.

There is scarcity of studies on point prevalence of both depressive and anxiety symptoms among adolescent women; however, some studies have examined this in the general population of perinatal women. Our findings align with those of Andersson et al. who reported a prevalence of 29.2% in depressive symptoms among a general population of women in Sweden during pregnancy, which declined to 16.5% at six months postpartum [9]. In their systematic review on the prevalence of depression across different pregnancy trimesters, Bennett et al. reported a prevalence of 12.8% during the second trimester and 12% during the third trimester [8]. The prevalence of depressive symptoms among our adolescent mothers at the third trimester was similar to that reported by Bennet et al. at the third trimester. A study conducted in Mexico that examined depressive symptoms at different times during pregnancy and post-partum reported slightly higher prevalences than the current study; a prevalence of 16.6% was reported at the second week of pregnancy, which increased to 17.1% at six weeks, and 20.0% at six months postpartum [11]. Factors that could account for the observed differences in point prevalence of depressive symptoms among studies include sociodemographic and socio-cultural differences among study populations, differing times when depressive symptoms were measured during pregnancy and post-delivery, differences in the instruments used to assess depression and difference in cut-off points on the screening tools used to classify participants as displaying depression [38, 39].

To the best of our knowledge no previous research has examined the point prevalence of anxiety at different times among the general population of perinatal women or among adolescent mothers. Moreover, all previous studies that have investigated point prevalence of depression in perinatal women have examined it in the general population of pregnant women; whereas, the present study examined point prevalence in pregnant adolescent women. This addresses a significant knowledge gap in the research literature.

We are also not aware of any studies in the research literature that have investigated the incidence of both depressive and generalized anxiety symptoms among perinatal adolescents in Tanzania. It is notable that the increasing incidence in depressive symptoms seen in the adolescents who participated in the present study differed from that reported in a general population of perinatal women in Mexico. In contrast to our study, the Mexican study found an incidence of depressive symptoms of 11.4% at six weeks gestation, which decreased to 9.0% at six months post-delivery [11]. The difference between our findings and those observed in the study conducted in Mexico could be due to the different populations of women who participated in the studies (i.e., general population of pregnant women versus pregnant adolescent women) and the times that depressive symptoms were measured (i.e., 2nd trimester to three months post-delivery versus 1st trimester to six months post-delivery).

Despite the incidence of both depressive and anxiety symptoms increasing from recruitment in the second trimester of pregnancy to 3 months postdelivery, we observed that the point prevalence of depressive and anxiety symptoms decreased over this time period. A possible explanation for this decrease in the point prevalence of depressive and anxiety symptoms is that the adolescent women who participated in the present study were involved in a non-pharmacological intervention involving Cognitive Behavior Therapy (CBT) from pregnancy to three months post-delivery. Hence, the intervention could have resulted in a decrease in point prevalence of depressive and/or anxiety symptoms. It is notable that the incidence of both depressive and generalized anxiety symptoms was consistent and high across all time periods. This is of specific public health significance and supports the contention that screening should be done earlier and repeatedly to detect new cases.

Sociodemographic differences among adolescent women with and without depression and/or anxiety symptoms revealed that marital status and partner violence were associated with depressive symptoms during pregnancy and to 3 months post-delivery, while marital status and planned pregnancy were associated with anxiety symptoms during pregnancy and to 3 months post-delivery. These associations have been observed and reported in previous studies [15, 16, 38, 40, 41]. Marriage and planned pregnancy have been documented to enhance psychological well-being, reducing vulnerability to stressors and hence be protective of depression and anxiety risk during pregnancy and post-delivery [42]. However, in contrast to previous research, in the present study, participants who were married had higher odds for depressive symptoms post-delivery. Adolescent motherhood is associated with risks related to the transition to adulthood and parenthood. These include physical changes, potential health complications, and often lack adequate knowledge, skills, and resources to cope with early motherhood [43] which may elevate stress levels in adolescent mothers and make them more vulnerable to mood disorders [44].

## Strengths

We recruited a large sample of adolescent women from antenatal clinics. The attrition rate for the present sample was 3.4%, which was very low, reducing the potential effects of bias due to loss to follow-up. We presented the first data on point prevalence and incidence related to anxiety among pregnant women and the first data on point prevalence and incidence related to both depression and anxiety in adolescent mothers in Tanzania, though there might be unpublished research.

## Limitations

The present study also has some limitations. First, screening measures were used to assess depressive and anxiety symptoms; no structured diagnostic interview was conducted to diagnose depression or anxiety in participants. These screening measures required that participants recall how they had been feeling in the past two weeks, which may have resulted in recall bias and affected the accuracy of the estimated prevalences. Also, the study was conducted in urban health facilities in Mwanza, Tanzania; therefore, the estimated prevalences of depression and anxiety may not be generalizable to rural settings. Given the lack of adjustment for multiple testing, findings should be considered exploratory and interpreted with caution regarding potential Type I error. Further, we used repeated assessment of depressive and anxiety symptoms at each time point using the same measures, the EPDS and the GAD-7. This could have resulted in measurement artifacts. Also, the use of a convenience sampling approach from the selected clinics may limit the representativeness of findings. Finally, this study was conducted within the context of an intervention. The intervention may have affected the estimates and therefore to obtain unbiased estimates of prevalence and incidence, future assessments should be conducted under routine care conditions. Having said this, we hope that screening and intervention strategies such as those employed do become a part of routine care. To facilitate this, future research should consider conducting randomized controlled trials of the intervention, utilize diagnostic measures of depression and anxiety, expand the intervention to rural settings, and consider the use of random sampling to maximize representation and generalizability. The sample size calculations in this study were precision-based and used prevalence rather than incidence estimates in the calculations. As such, the sample size may have been more appropriate for prevalence than incidence estimation. However, as the standard error of an estimated proportion tends to be higher for larger proportions, and is maximized at 50%, this did not lead to issues of imprecision for our incidence estimates.

## Conclusions

Depressive and generalized anxiety symptoms increased in incidence from second trimester of pregnancy to three months post-delivery, but a decrease in point prevalence in adolescent women who participated in a non-pharmacological intervention. These findings support the importance of integrating mental health services into existing antenatal and postnatal care services in Tanzania. They also bring to the attention of authorities and policy makers the importance of delivering routine mental health services as early as possible to adolescent mothers during the perinatal period. Targeted marital and partner-violence interventions should be offered as part of general maternal mental health services.

## Data Availability

The datasets used and/or analyzed during the current study are available from the corresponding author on reasonable request.
