# Correction: Point prevalence and incidence of depressive and generalized anxiety symptoms among women attending antenatal clinics, a longitudinal study among adolescent mothers in Mwanza Tanzania

**DOI:** 10.1186/s13034-026-01052-1

**Published:** 2026-02-21

**Authors:** Matiko Mwita, Scott Patten, Deborah Dewey, Eveline T. Konje

**Affiliations:** 1 Psychiatry Department, Catholic University of Health and Allied Sciences (CUHAS), P. O. Box 1464, Mwanza, Tanzania; 2 Psychiatry Department, Bugando Medical Centre (BMC), Mwanza, Tanzania; 3 Cuthbertson & Fisher Chair, Departments of Community Health Sciences and Psychiatry, University of Calgary, Calgary, Canada; 4 Departments of Pediatrics and Community Health Sciences, University of Calgary, Owerko Centre at the Alberta Children’s Hospital Research Institute, Hotchkiss Brain Institute, Calgary, Canada; 5 Department of Epidemiology and Biostatistics, Catholic University of Health and Allied Sciences (CUHAS), Mwanza, Tanzania

**Correction: Child and Adolescent Psychiatry and Mental Health (2025) 19:122**

10.1186/s13034-025-00983-5

In the original publication of this article [1], the author identified the following errors. In Results section, the prevalence values are correctly presented as percentages. Their corresponding 95% confidence intervals are mistakenly reported in decimal proportion format instead of percentage format (pages 1, 4, and 5).

In Table 2 (page 5), Table 4 (page 6) and associated descriptions (pages 4–7), some odds ratios (ORs) fall outside their correscponding 95% Confidence Intervals (CIs).

In Abstract under Results section, the correct version should read, “The point prevalence of both depressive and generalized anxiety symptoms decreased from the second trimester of pregnancy (T1) to 3 months post-delivery (T4). The point prevalence of depressive symptoms fell from 20.64% (95% CI, 17.02–24.61) at T1 to 9.90% (95% CI, 7.18–12.52) at T4, while the point prevalence of generalized anxiety symptoms fell from 22.33% (95% CI, 19.42–26.18) at T1 to 10.48% (95% CI, 8.15–13.04) at T4. In contrast, the incidence of both depressive and anxiety symptoms increased from recruitment (T1) through to 3 months post-delivery. Specifically, the incidence of depressive symptoms rose from 9.00% (95% CI, 7.12–12.47) at T2 to 11.89% (95% CI, 7.13–12.67) at T4, while the incidence of generalized anxiety symptoms rose from 7.20% (95% CI, 5.16–10.12) at T2 to 10.81% (95% CI, 6.17–11.51) at T4. At all-time points, being classified as displaying depressive symptoms was highly associated with being classified as displaying symptoms of anxiety”.

In Results section under sub-heading “Point prevalence and incidence of perinatal depressive and anxiety symptoms”, the paragraphs should read, “The point prevalence of perinatal depressive symptoms (EPDS≥9) was 20.64% (95% CI, 17.02–24.61) in the second trimester of pregnancy (T1), 13.37% (95% CI, 11.72–16.41) in the third trimester of pregnancy (T2), 14.37% (95% CI, 11.01–17.34) at four weeks post-delivery (T3) and 9.90% (95% CI, 7.18–12.52) at three months post-delivery (T4). The incidence of perinatal depressive symptoms at T2 (n = 421) was 9.00% (95% CI, 7.12–12.47), at T3 (n = 367) it was 9.80% (95% CI, 6.12–12.42) and at T4 (n = 328) it was 11.89% (95% CI, 7.13–12.67) (Table 2). The observed incidence increased by 0.8% from T2 to T3 and by 2.09% from T3 to T4.

The point prevalence of generalized anxiety symptoms (GAD-7 ≥5) was 22.33% (95% CI, 19.42–26.18) in the second trimester of pregnancy (T1), 12.43% (95% CI, 11.01–15.40) in the third trimester of pregnancy (T2), 9.90% (95% CI, 7.31–12.17) at four weeks post-delivery (T3) and 10.48% (95% CI, 8.15–13.04) at three months post-delivery (T4). The incidence of generalized anxiety symptoms at T2 (*n* = 412) was 7.20% (95% CI, 5.16–10.12), at T3 (*n* = 366) it was 9.02% (95% CI, 6.12–11.61) and at T4 (*n* = 333) it was 10.81% (95% CI, 6.17–11.51) (Table 2). The observed incidence increased by 1.74% from T2 to T3 and by 1.79% from T3 to T4.”

In Table 2, the corrected values are given below:

- 95% CI for Point prevalence of depressive symptoms:
  - At Time 1, correct value should read: 17.02–24.61.
  - At time 2, correct value should read: 11.72–16.41.
  - At time 3, correct value should read:11.01–17.34.
  - At time 4, correct value should read: 7.18–12.52.
- 95% CI for incidence of depressive symptoms:
  - At time 2, correct value should read: 7.12–12.47.
  - At time 3, correct value should read: 6.12–12.42.
  - At time 4, correct value should read: 7.12–12.67.
- 95% CI for Point prevalence of anxiety symptoms:
  - At Time 1, correct value should read: 19.42–26.18.
  - At time 2, correct value should read: 11.01–15.40.
  - At time 3, correct value should read:7.31–12.17.
  - At time 4, correct value should read: 8.15–13.04.
- 95% CI for incidence of anxiety symptoms:
  - At time 2, correct value should read: 5.16–10.12.
  - At time 3, correct value should read: 6.12–11.61.
  - At time 4, correct value should read: 6.17–11.51.

In Table 4, the corrections are mentioned below:

- At Time 4, for participants who were married, the correct value should read: 6.9(1.4–7.5).
- For participants living with parents/care takers:
  - At Time 1, the correct valuen should read: 10.3(1.5–15.3).
  - At Time 4, the correct value should read: 4.9(1.2–6.5).

Under sub-section “Sociodemographic differences among adolescent women with and without anxiety symptoms”, the first paragraph should read, “Associations between sociodemographic variables and anxiety symptoms also differed by time (see Table 4). In the second trimester of pregnancy, (Time 1), those who were married (OR = 0.7, 95% CI: 0.1,0.8, p = 0.043), and those who planned for their pregnancies (OR = 0.4, 95% CI:0.3,0.8, p = 0.005) had significantly lower odds of anxiety symptoms. Those living with parents/care takers (OR = 10.3, 95% CI: 1.5,15.3, p = 0.016), had increased odds for anxiety symptoms. Experiencing partner violence was associated with significantly increased odds for anxiety symptoms during pregnancy (Time 1: OR = 4.6, 95% CI: 2.1,9.9, p < 0.001, Time 2: OR = 3.0, 95% CI: 1.3,6.8, p = 0.007). Those who planned for their pregnancies showed significantly decreased odds for anxiety symptoms post-delivery (Time 3: OR = 0.4, 95% CI: 0.2,0.8, p = 0.012, Time 4: OR = 0.5, 95% CI: 0.2,0.9, p = 0.022). At Time 4 (three months postpartum), those who were married (OR = 6.9, 95% CI: 1.4,7.5, p = 0.018) and those living with parents/care takers (OR = 4.9, 95% CI: 1.2,6.5, p = 0.047) showed significantly increased odds for anxiety symptoms. Age, education level and employment status were not associated with anxiety symptoms during pregnancy or post-delivery”.
